# 4054 Telemedicine Infectious Diseases Consultation in Rural Hospitals: Feasibility, Acceptability, Appropriateness, and Implementation

**DOI:** 10.1017/cts.2020.430

**Published:** 2020-07-29

**Authors:** Jason P. Burnham, Stephanie Fritz, Graham Colditz

**Affiliations:** 1 Washington University in St. Louis, Institute of Clinical and Translational Sciences

## Abstract

OBJECTIVES/GOALS: The objective of this study is to examine implementation science and clinical outcomes of telemedicine ID consultation at a rural Missouri hospital. METHODS/STUDY POPULATION: Pilot study, hybrid type 2, studying clinical outcomes (mortality, readmission, hospital transfer) and implementation outcomes assessed by survey and chart review (feasibility, acceptability, appropriateness, fidelity to guideline-based care). Telemedicine ID consultations are carried out for patients at Missouri Baptist Sullivan Hospital (MBSH) with positive blood cultures and charts reviewed for 30 days after hospital discharge. Patients, physicians, and staff complete surveys for implementation outcomes. The practical, robust implementation and sustainability model (PRISM) was chosen as the framework for this study and its future scale-up. RESULTS/ANTICIPATED RESULTS: There were 46 patients with positive blood cultures at MBSH, 20 of which were transferred or left from the ER before consultation could be offered. Eighteen patients had telemedicine ID consultation. The remaining 8 patients had contaminants in their blood cultures and therefore no consultation was offered. Of eligible patients not transferred, recruitment rate was 100% (18/18). Average total time per consult was 52.8 minutes on day 1, 8.5 minutes on day 2. 30-day mortality was 0%, 30-day readmission rate 5.5% (n = 1), hospital transfer rate 5.5% (n = 1). 13 patients and 9 providers completed the feasibility, acceptability, and appropriateness survey with zero negative responses on any measure. DISCUSSION/SIGNIFICANCE OF IMPACT: Telemedicine ID consultation at a single rural hospital has thus far been received as feasible, acceptable, and appropriate. Scale-up of this model of care remains to be studied.

